# Quantification Methods for Textile-Adhered Bacteria: Extraction, Colorimetric, and Microscopic Analysis

**DOI:** 10.3390/polym11101666

**Published:** 2019-10-12

**Authors:** Tahmineh Hemmatian, Jooyoun Kim

**Affiliations:** 1 Department of Textiles, Merchandising and Fashion Design, Seoul National University, Seoul 08826, Korea; hanh11@snu.ac.kr; 2 Research Institute of Human Ecology, Seoul National University, Seoul 08826, Korea

**Keywords:** textile, *E. coli*, adhesion, extraction, colorimetric, microscopy

## Abstract

Quantification of bacteria adhered on porous, multi-layered fibers is a challenging task. The goal of this study is to compare different assessment procedures on counting textile-adhered bacteria, and to guide relevant analytical techniques. Three different methods were compared in measuring the amount of *Escherichia coli* (*E. coli*) adhered to polymeric film and fibrous nonwovens. In the extraction method, the adhered bacteria were released with the assistance of surfactant/enzyme, where the measurement was rather reproducible. For colorimetric method, stained bacteria enabled direct visualization without needing to detach cells from the surface, yet the linearity of color absorbency to cell counts was limited. The microscopic analysis provided direct observation of bacterial distribution over the surface, but accurate quantification was not possible for porous, fibrous surfaces. This study intends to help choosing a suitable test method to accurately quantify the textile-adhered bacteria, as well as broadly impact the research on anti-bioadhesive surfaces.

## 1. Introduction

Bacterial adhesion [1,2,3,4] and biofilm formation on material surfaces not only limit the material performance but also are liable for health risks [4,5,6]. For textile materials, bacteria can adhere to fibrous surfaces during wear, and its adhesion is likely to cause malodor and bacterial infection. To prevent the bacterial adhesion and growth on materials, antimicrobial treatments using biocidal nanoparticles or antibiotics are frequently done [7,8,9,10]. Among the biocidal nanoparticles, silver nanoparticles have often been applied to a wide range of materials due to their efficacy at interfering with bacterial metabolism [11,12,13,14]. However, the influence of nanoparticles and the reactive species on human cells is not fully understood [15,16,17], and this limits the application of silver nanoparticles [18,19,20,21]. 

In addition, with the evolvement of antibiotic-resistant bacteria, concerns on abusive use of antibiotics have been addressed. Thus, material design that resists the bacterial adhesion to surfaces has been explored as an alternative measure to the usage of biocides [22,23,24]. For the anti-bioadhesive properties, numerous physical aspects of materials have been considered, including surface energy [25,26], wettability [26,27,28,29], surface charge, and topography [30,31,32,33]. Among those parameters, wetting property has been often associated with the microbial adhesion. However, the wetting criteria beneficial for bacterial anti-bioadhesion are not conclusive; some studies reported that super-hydrophobic surfaces (water contact angle > 150°) is advantageous in circumventing bacterial adhesion [30,32,33], and others reported that a hydrophilic surface is desirable for reducing the bacterial adhesion [31,34].

While there have been numerous studies on surface interaction with bacterial adhesion [6,25,31,35,36], little studies are available for validating the experimental methods for bacterial adhesion on surfaces [31,37]. As the interpretation of bacterial adhesion depends on the evaluation methods, validation of relevant method is a pre-requisite for the research on anti-bioadhesive surfaces. The complex 3D structure of fibrous materials, in particular, makes the quantification of adhered bacteria even more challenging, thus making a proper evaluation method necessary for accurate evaluation of textile-adhered bacteria. In previous studies, microscopic analysis has been commonly employed to visually observe the adhered bacteria on surfaces [31,38,39,40,41,42]. However, it is difficult to accurately count the bacteria in the depth layers of fibrous materials [31]. Staining may enhance the visibility of bacterial presence, but common dyes may stain not only the bacteria but also the material itself, resulting in high background signals [43,44]. Fluorescence labeling of bacteria is a convenient way of visualizing the bacteria [42,45,46,47,48,49,50,51], but careful adjustment of light exposure should be made to minimize the influence of auto-fluorescence of polymeric background [31,52]. 

Extraction method [53,54,55] that releases the adhered bacteria prior to measuring optical density or counting the colony forming units (CFU) is a commonly applied quantification method but it may lack the precision if the bacteria detachment from the surface is incomplete [56,57]. For precise quantification, metabolic activity of viable bacteria can be detected, but it requires sophisticated technique and materials. Recently, optical visualization method using iodonitrotetrazolium (INT) chloride was suggested as a cost-effective quantification method for textile-adhered bacteria, where INT changes color to purple upon capturing two electrons from viable bacteria [43]. Yet it lacks thorough validation in comparison with others.

The objective of this study is to compare different evaluation techniques applied to textile-adhered bacteria and to help in selecting a facile and relevant analytical method. Two types of fibrous substrates with different fiber diameters and layer depths were used, which were polypropylene (PP) meltblown and polyethylene terephthalate (PET) spunbond (SB) nonwovens. In addition, as a comparison, PET film with a smooth surface was tested. Experimental validation was conducted using *Escherichia coli (E. coli)* bacteria, which has an increasing relevance due to their public health risks resulting from nosocomial infections and antibiotic resistance profiles. For quantitative analysis of adhered bacteria, three different methods were tested: (1) extraction of textile-adhered bacteria, with the subsequent optical density measurement and CFU counting; (2) colorimetric analysis by INT staining of live textile-adhered bacterial cells; and (3) microscopic analysis for calculation of the area fraction covered by bacteria. Each quantitative measurement was comparatively evaluated in correlation with actual CFU or adhered area.

Developing a proper quantification method for textile-adhered bacteria requires a collective understanding of both bacteria and material. Through the experimental validation of different methods, it is anticipated to identify a suitable test method that can efficiently measure the bacterial-adhesive properties for specific surfaces and analytical situations. While this study uses *E. coli* for method optimization, it is thought that the overall methodological procedures and verification processes can be expanded to other bacteria. This study intends to broadly impact the research in the field of anti-bioadhesive surfaces and related product design.

## 2. Materials and Methods 

### 2.1. Materials

Polyethylene terephthalate films (PET-F) were purchased from Goodfellow (Huntingdon, UK). PET spunbond (PET-SB) and polypropylene meltblown (PP-MB) nonwovens were supplied from Korea Institute of Industrial Technology (KITECH, Ansan-si, Gyeonggi-do, Korea). The solidity and porosity of the webs were calculated by Equations (1) and (2). The mean diameter of nonwoven fibers were measured from the scanning electron microscopy (SEM) images. The characteristics of the film and nonwovens used in this study are shown in Table 1.

Solidity (unitless) = m/(A·t·ρ),(1)

Porosity (%) = [1 − solidity] × 100 (%),(2)

where m (g) is the sample mass; A (${\mathrm{cm}}^{2}$) is the sample area; t (mm) is sample thickness; and ρ (g/${\mathrm{cm}}^{3}$) is the polymer density (1.35 g/cm^3^ for PET, 0.91 g/cm^3^ for PP).

*E. coli* strain KCTC 1039 was used as the test bacteria. Phosphate buffered saline (PBS) and the TrypLE Express enzyme for cell detachment was purchased from Thermo Fisher Scientific (Waltham, MA, USA). PVDF microfilters in 0.22 μm pore size were purchased from BIOFIL (Guangzhou, China), and dimethyl sulfoxide (DMSO) was purchased from Daihan Scientific (Wonju-si, Gangwon-do, Korea). Sodium dodecyl sulfate (SDS), iodonitrotetrazolium (INT) chloride culture Luria-Bertani broth (LB), and all other chemicals were purchased from Sigma-Aldrich (St. Louis, MO, USA). 

### 2.2. Adhering Bacteria to Substrates

**Figure 1:**
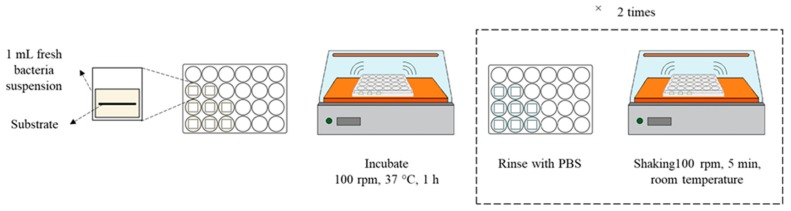
Procedure of adhering bacteria to substrates. PBS = phosphate buffered saline.

Prior to the test, sample substrates (1 cm × 1 cm) were cleaned in isopropanol for 5 min, using a sonicator (Digital Ultrasonic Cleaner, WUC-D03H, Daihan Science Co., Gangwon-do, Korea), and were then rinsed with distilled water. For bacterial binding to a sample substrate, the substrate was immersed in a 1 mL of bacterial culture in LB broth, with the initial OD_600_ of 0.5 that corresponds to 3.4 × 10^8^ cells/mL. The 1 mL of culture and a substrate were put in a 24-well plate and incubated for 1 h at 100 rpm. After incubation, the *E. coli*-adhered substrate was placed in a new plate; then the weakly adhered bacteria were removed from the sample by rinsing the substrate two times in 1 mL PBS at 100 rpm for 5 min. The bacterial binding procedure is illustrated in Figure 1.

### 2.3. Evaluation Methods for Bacterial Adhesion on Polymeric Substrates

For evaluation of surface-adhered bacteria on polymeric substrates, three different methods were tested as follows: Method 1, extraction of surface-adhered bacteria and CFU counting; Method 2, INT-staining of bacteria and colorimetric analysis; and, Method 3, microscopic analysis. Detailed experimental procedures are detailed in the following.

#### 2.3.1. Method 1: Extraction of Surface-Adhered Bacteria and CFU Counting

Standard Curve for Optical Density and Cell Counting: *E. coli* in LB broth was incubated at 37 °C and 200 rpm for 4 h; then the bacteria suspension was pelleted by centrifuging at 3000 rpm for 5 min at 4 °C (Multi Centrifuge-VARISPIN 15R, CRYSTE, Bucheon-si, Gyunggi-do, Korea). The supernatant was removed, and the pellet was resuspended in PBS to the OD_600_ of approximately 0.5 that corresponds to 3.4 × 10^8^ cells/mL, from which a series of diluted suspension was prepared.

**Figure 2:**
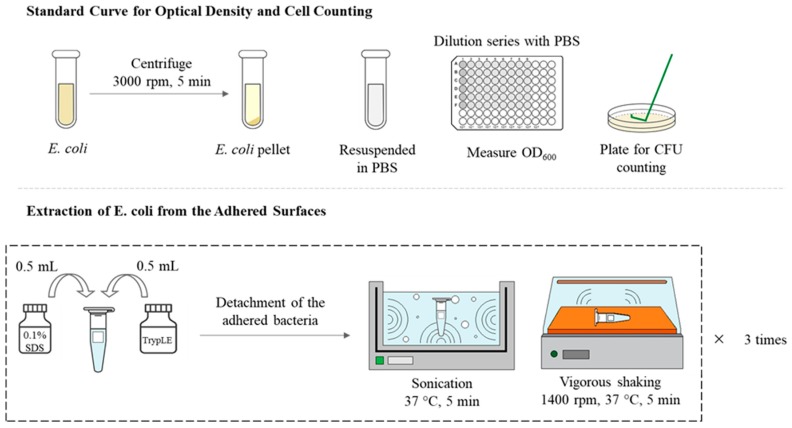
Method 1, extraction of surface-adhered bacteria and cell counting. CFU = colony forming units.

The optical density at 600 nm wavelength (OD_600_) of the dilution series was measured using a microplate reader spectrophotometer (SpectraMax 190, Molecular Devices LLC, San Jose, CA, USA). The optical OD_600_ of the suspension was corrected for a blank PBS solution. A 20 μL of each dilution was plated on a LB agar for cell counting, and the OD_600_ of dilution series was correlated with the cell counting in cells/mL. Figure 2 illustrates the procedures for standard curve generation and cell extraction.

Extraction of E. coli from the Adhered Surfaces: For the full extraction of bacterial from the textile materials, the extraction methods used in the previous studies [53,54,55] were modified for centrifugation speed, use of surfactant/enzyme, etc. The *E. coli*-adhered surface was prepared as detailed in the previous section. After removing the weakly adhered bacteria by gentle rinsing procedure, the remaining bacteria on surface were extracted using a surfactant/enzyme solution, and the OD_600_ of the extract was measured to estimate the amount of released bacteria. For detachment of bacteria, the bacteria-adhered substrate was placed in a centrifuge tube containing 0.5 mL of 0.1% sodium dodecyl sulfate (SDS)/PBS solution and 0.5 mL of TrypLE Express enzyme; then the solution was sonicated in an ultrasonic bath for 5 min at 37 °C (60 Hz with the power output of 300 W).

After sonication, tubes were shaken at 1400 rpm for 5 min at room temperature, using a micro mixer (Thermo micro mixer Mxi4t, FINEPCR, Gunpo-si, Gyeonggi-do, Korea). Each sample was underwent the extraction procedures three times, using a fresh solution with SDS surfactant and TrypLE Express enzyme. Three extracts with detached cells were combined for measurement of OD_600_. The number of CFU was correlated with the OD_600_ measurement and expressed as cells/mL solution or cells/cm^2^ substrate surface.

#### 2.3.2. Method 2: INT-staining of Bacteria and Colorimetric Analysis

*Standard Curve for Color Absorbency and Cell Counting:* A 9.9 × 10^−3^ M of INT stock solution was prepared in PBS. One mL of bacteria suspension in PBS was transferred to a tube and a 200 μl (1.98 × 10^−6^ moles) of the prepared INT stock solution was added to the suspension, followed by the incubation with 100 rpm for 4 h at 37 °C. During the incubation, the tetrazolium salt was reduced by accepting the electrons of active cells, and formazan was formed. The formazan-formed culture suspension was pelleted at 14,000 rpm for 30 min. The colorless supernatant was then removed, and the pelleted formazan in red color was resuspended in a 2 mL of DMSO by sonicating for 2 min to extract the formazan (colorant) to DMSO. For a thorough transfer of formazan to DMSO, the suspension was heated to around 105 °C for 5 min. The suspension solution was then filtered through a micro filter (0.22 μm pore), and dilution series of INT-DMSO eluent was prepared for absorbency measurement at the wavelength of 470 nm using a spectrophotometer.

**Figure 3:**
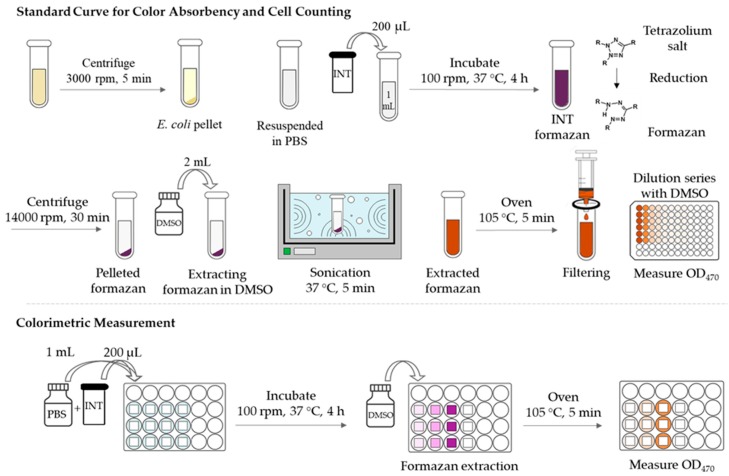
Method 2, colorimetric analysis with iodonitrotetrazolium (INT) staining of cells. DMSO = dimethyl sulfoxide.

*Colorimetric Measurement:* To estimate the number of adhered bacteria that contributed to formazan formation, the bacteria-adhered substrates were incubated with 100 rpm for 4 h at 37 °C, in a 24-well plate containing 200 μL (1.98 × 10^−6^ moles) of prepared INT stock solution and 1 mL of PBS. After incubation with INT, samples were moved to a new plate and the formazan was extracted with a 2 mL of DMSO; the suspension in DMSO was heated to about 105 °C for 5 min for a complete extraction. The suspension was then filtered through a microfilter (0.22 μm pore), and the INT formazan/DMSO eluent was measured for its absorbency at the wavelength of 470 nm using a spectrophotometer. The number of adhered bacteria was correlated with OD_470_ measurement and expressed as CFU/mL solution or CFU/cm^2^ substrate surface. Figure 3 illustrates the procedure for the colorimetric measurement.

#### 2.3.3. Method 3: Microscopic Analysis

Scanning electron microscopy (SEM) imaging of bacteria-adhered substrates on PP-MB, PET-SB, and PET-F, with and without extraction procedure (method 1), was analyzed. The bacteria-adhered substrates were fixed with 2% (*v*/*v*) glutaraldehyde in PBS (pH 7.4) at around 23 °C for 2 h, then exposed to the vapor of 2% (*w*/*v*) osmium tetroxide for 2 h. The treated samples were sputter-coated with platinum in about 5 nm thickness (20 mA, 180 sec sputter time) prior to SEM analysis (JSM-5410LV, JEOL Ltd, Tokyo, Japan).

### 2.4. Statistical Analysis 

Statistical analysis was performed using SPSS v.25.0 to test whether the measured values among the samples are significantly different. The one-way ANOVA and the pairwise comparison were performed using the Bonferroni correction. The *p*-value was examined for the significance level. 

## 3. Results and Discussion

### 3.1. Method 1: Extraction Method

**Figure 4:**
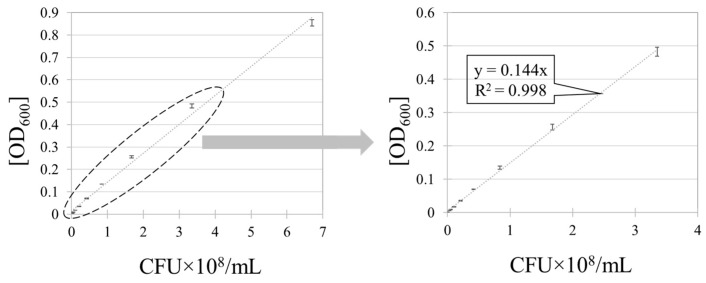
Method 2: OD_600_ of *Escherichia coli* (*E. coli*) suspension and CFU. Error bars represent the standard deviations of three individual replicates.

The fitted line in Figure 4 was produced by the cell count of serial dilutions. The OD_600_ of *E. coli* suspension was linearly correlated with the CFU. The actual measurements of surface-adhered bacteria were made in the lower bacterial concentration range, thus correlating this lower range for the accurate prediction of surface-adhered bacteria.

In this method, bacteria-adhered surface was extracted with a surfactant solution formulated with SDS surfactant and TrypLE enzyme for an effective detachment of bacteria cells. The enzyme is known to cleave the peptide bonds on the C-terminal side of lysine and arginine, cutting the cell-matrix interactions and leading to the cell rounding. During this rounding process, the position and integrity of the adhesion sites are maintained, while the cell detachment process is facilitated [58]. The SDS, an anionic surfactant, plays a role in disrupting the ionic bonds in proteins [59]. The enzyme and SDS, together with the mechanical agitation by sonication and shaking, helped detachment of surface-adhered bacteria. 

**Figure 5:**
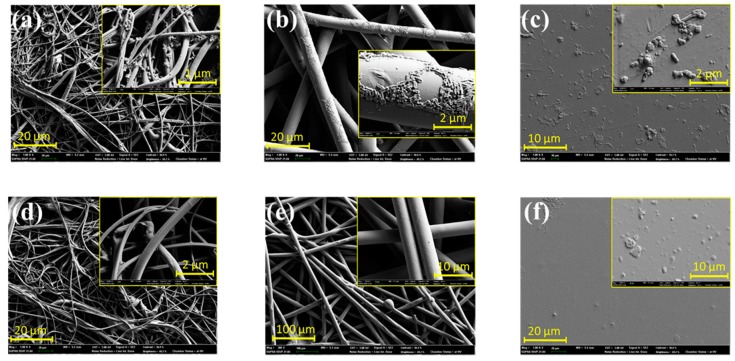
SEM image of substrates before and after extraction. (**a**) PET-MB before extraction; (**b**) PET-SB before extraction; (**c**) PET-F before extraction; (**d**) PET-MB after extraction; (**e**) PET-SB after extraction; and (**f**) PET-F after extraction (particles in the inserted image are not bacteria).

Figure 5 shows the *E. coli*-adhered surfaces prior to (Figure 5a–c) and post extraction (Figure 5d–f). As the images indicate, surfaces after the extraction appeared to be free of bacteria. For the flat surface, such as PET-F, it was obviously shown that the extraction procedure successfully released the adhered bacteria. Multi-layered fibrous structures, such as PET-SB and PP-MB, while the depth-layers were not observable, also displayed bacteria-free surface. 

**Figure 6:**
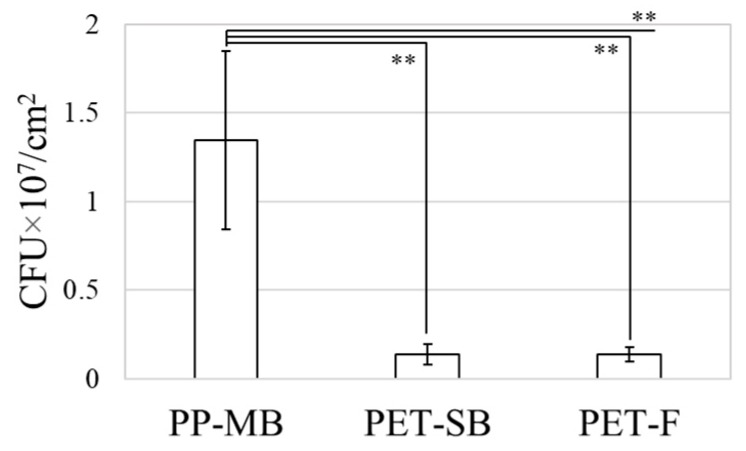
Amount of bacteria extracted from substrates. Error bars represent the standard deviation of three individual textile samples. (** *p* < 0.01).

The released *E. coli* from the surface was measured at OD_600_, and the CFU was correlated as the CFU per unit area in Figure 6. The PP-MB showed the highest number of adhered bacteria with large variations, as bacteria resided in depths of fibrous structure (Figure 5a). From Figure 6, PP-MB showed higher bacterial adhesion than PET-SB or PET-F. PP-MB is comprised of smaller fibers and greater fiber depths than PET-SB, providing more of micro-pores in depth fiber layers; this would increase the probability that bacteria adhere and get stuck in the depth layers. As a result, PP-MB had the greater number of bacteria adhered on the surface. The variation of the measured values for PP-MB was large, due to the irregularity of fiber surfaces of meltblown web. Due to the layer depth and irregularity of PP-MB, more bacteria were attached to the surface with a large variation. While it was not conclusive that the adhered bacteria were completely released from the fibrous surfaces, the results showed differences among the tested substrates. Based on the ANOVA test, there were significant differences in the number of adhered bacteria among the tested substrates (*p*-value = 0.004). Further pairwise comparison, with the Bonferroni correction, revealed that the mean number of adhered bacteria was significantly higher for PP-MB than PET-SB (*p*-value = 0.007), and for PP-MB than PET-F (*p*-value = 0.007). 

### 3.2. Method 2: Colorimetric Analysis with INT Staining

**Figure 7:**
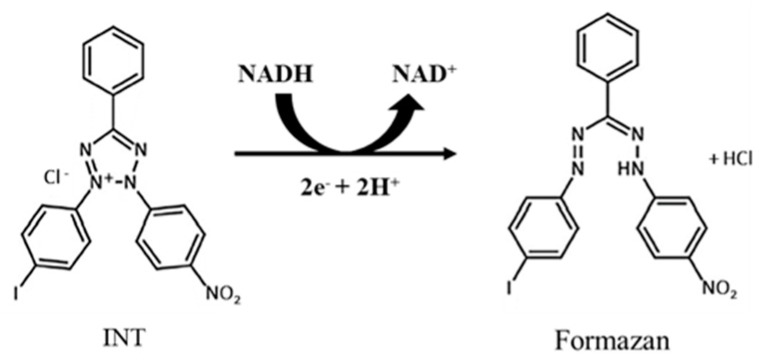
Reaction of tetrazolium reduction and formazan production in INT.

In a few earlier studies, the colorless iodonitrotetrazolium (INT) salt was used to measure the microbial viability [43,60], as INT transforms into purple formazan by reacting with live cells through the reduction process (Figure 7). Nicotinamide adenine dinucleotide (NAD), a coenzyme found in all living cells, exists in two forms: NAD+ (oxidized form) and NADH (reduced form), where NADH of live cells can act as a reducing agent to donate electrons to INT [61], forming a purple formazan. The formazan is insoluble in water and forms crystals on the location where the INT captures electrons in the cell [44]. Those crystals are easily solubilized in DMSO, and the colorant solution produces a distinctive absorbency peak at 470 nm [OD_470_]. 

**Figure 8:**
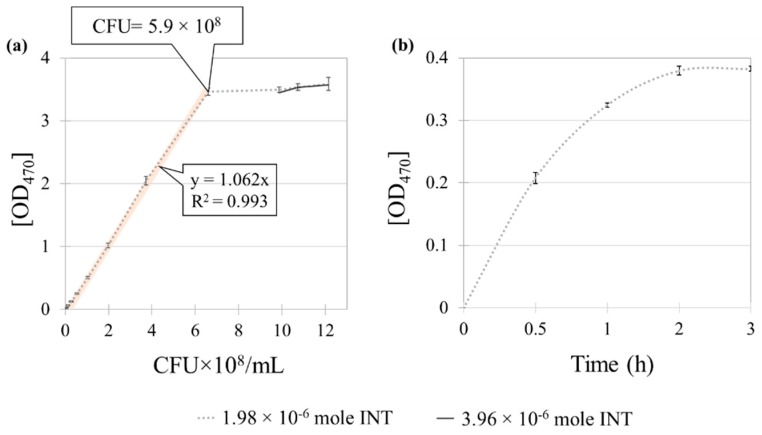
Relationship of OD_470_ and CFU for different INT concentration and incubation time. (**a**) OD_470_ and CFU with different INT concentrations; (**b**) INT absorbency (1.98 × 10^−9^ mole INT) with different reaction time. Error bars represent the standard deviation of three individual replicates.

To investigate whether the absorbency of formazan is in direct correlation with the number of viable bacteria, a fitted line was generated by correlating the OD_470_ with CFU (Figure 8a). When reacting with INT of 1.98 × 10^−6^ moles (200 μl of 9.9 × 10^−3^ M), a linear increase of absorbency with the viable cell numbers was observed up to about 6.6 × 10^8^ CFU, and thereafter the OD_470_ reached a plateau. To examine whether the additional amount of INT affects the color absorbency over CFU~ 6.6 × 10^8^, the INT amount was doubled (3.96 × 10^−6^ moles) to react with bacteria suspensions of 8.3 × 10^8^ cells/mL (OD_600_ ~ 1.2), 9.7 × 10^8^ cells/mL (OD_600_ ~ 1.4) and 11 × 10^8^ cells/mL (OD_600_ ~ 1.6), respectively. From Figure 8a, the OD_470_ with two different INT amounts overlapped for the tested suspensions, showing that 1.98 × 10^−6^ moles of INT were sufficient to react with bacteria cells. However, the linearity between OD_470_ and CFU was not met above the CFU~ 5.9 × 10^8^, thus determining the maximum detection limit of this method to be 5.9 × 10^8^ cells.

**Figure 9:**
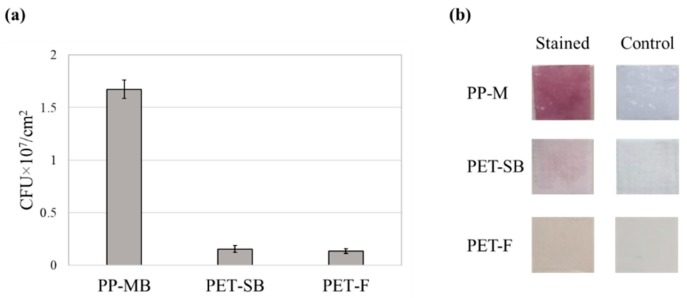
Measurement of substrate-adhered bacteria. (**a**) Quantification of CFU for different substrates; (**b**) visual observation of formazan (reduced INT) crystals formed on substrates. Error bars represent the standard deviation of three individual textile samples.

To determine the optimal reaction time for INT and bacteria, the bacterial suspension with 2.7 × 10^8^ CFU (1 mL of suspension with OD_600_ ~ 0.4) was reacted with 1.98 × 10^−6^ moles of INT for different reaction time, 0.5, 1, 2, 3 h, respectively (Figure 8b). The result showed that 2 h incubation time was sufficient to complete the reaction. To ensure the entire reaction of INT to form formazan, 4 h of incubation time was applied for later experiments. Based on the CFU-OD_470_ correlation, the quantity of bacteria adhered to different substrates was measured in Figure 9a. The amount of adhered bacteria was clearly visible by different color shades shown in Figure 9b. 

**Figure 10:**
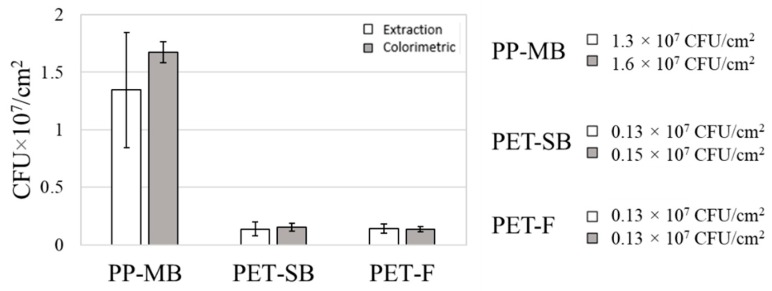
Comparison of extraction and colorimetric methods. Error bars represent the standard deviation of three individual textile samples.

The measured amounts of surface-adhered bacteria by methods 1 and 2 are compared in Figure 10. The amounts of bacteria adhered to PET-F and PET-SB were appeared nearly equal for both methods 1 and 2. As the SEM images after extraction (Figure 5) show, the extraction method successfully released the adhered bacteria from PET-F and PET-SB surfaces. As those surfaces were rather regular with little depth layers, the extraction method seemed to be effective in quantifying the adhered bacteria. However, the extraction may become less effective with longer incubation time with bacteria. The amount of adhered bacteria in PET-F and PET-SB showed significantly less than that of PP-MB. For PP-MB that had highly irregular 3D structure with fine fibers, the detachment of cells may not be as effective as the flat surfaces, leaving the cells remained on substrates. However, the colorimetric method and extraction method in this study produced similar results, and it was thought that the addition of SDS and enzyme helped in releasing most of adhered cells from the substrates. 

The colorimetric method produced reproducible results with smaller variations (more precise with less scatters) than the extraction method. As it is probable that the detachment of bacteria is incomplete by the extraction, method 2 of the colorimetric analysis would be likely to produce more accurate result in detecting the surface-adhered bacteria, as long as the linearity of OD_470_ and CFU is met. In the colorimetric method, INT reacts only with live cells, while the extraction may count both the live and dead cells in the suspension. Thus, the colorimetric method would be useful for investigating the active cells that are likely to be infectious or odor-forming.

### 3.3. Method 3: Microscopic Analysis

**Figure 11:**
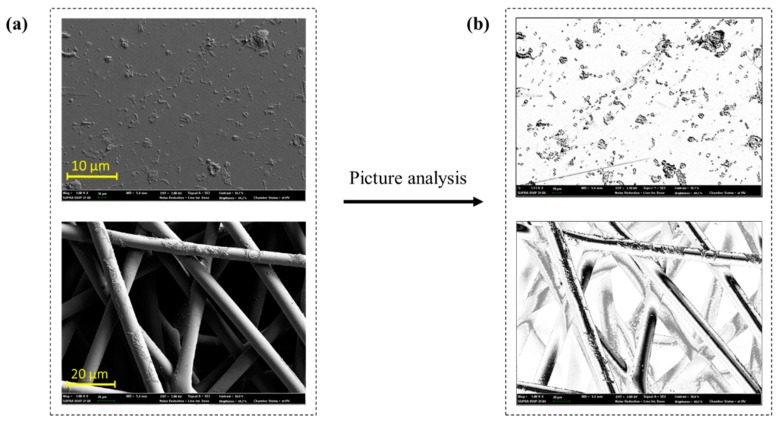
Image analysis for measurement of an area fraction (%) covered by *E. coli*: (**a**) SEM image of PET-F and PET-SB before extraction; (**b**) black and white binary image produced using Photoshop CS6.

For direct visualization, SEM images were analyzed for the area fraction (%) that was covered by the *E. coli* bacteria (Figure 11). In this method, the contrast of SEM image was processed to produce a binary image of black and white, then the pixels of distinctive contrasts were counted to calculate the % area fraction. As the contrast of SEM images are dependent on the height of surfaces, the contrast information from the fibrous surface did not accurately correspond with the bacterial presence. However, the bacteria on a flat surface, such as PET-F, was accountable to by this method. Table 2 shows the estimated number of bacteria adhered on PET-F surface by the image analysis; about 5.7 % of PET-F surface area was covered by *E. coli* after 1 h of bacterial incubation. 

### 3.4. Comparison of Methods 

Three different quantification methods were used for measuring the amount of adhered bacteria to monolayer (film) and multi-layer (fibrous nonwoven) surfaces. In the extraction method, where the CFU was linearly correlated with OD_600_, both live and dead cells are countable; however, complete detachment of bacteria cannot be assured, especially for porous and depth-layered substrates. For the colorimetric method, the cell detachment procedure was not necessary. In addition, the INT reaction occurred only for the live bacteria cells, thus preventing interference of the color signal with the background noise from the polymer staining. The direct visualization of bacterial presence was possible with the naked eye by the INT staining. The disadvantage of the colorimetric method is the limited detection range; however, the colorimetric produced a rather accurate results where the linearity between OD_470_ and CFU was met (CFU ≤ 5.9 × 10^8^). In microscopic analysis, direct observation of bacterial distribution on surfaces was possible, yet the accurate quantification was very limited, especially for porous, multi-layered surfaces. Three different quantification methods for surface-adhered bacteria are summarized in Table 3 for their relevancy and limitations.

This study compared different methods using the *E. coli*, which is the gram-negative bacteria. The gram-negative cells are composed of a hydrophobic lipopolysaccharide membrane, thus leading the interaction with the hydrophobic substrates to perhaps be stronger than the gram-positive cells that are composed of peptidoglycan. Thus, further study with the extended scope of materials and bacteria type are suggested for thorough analysis on the interaction between the cell type and the substrate chemistry. 

## 4. Conclusions

As the interpretation of bacterial adhesion depends on the evaluation methods, validation of a relevant method is a pre-requisite for research on anti-bioadhesive surfaces. In particular, the complex 3D structure of fibrous materials makes the quantification of adhered bacteria even more challenging, thus requiring the analytical technique to be validated for its relevancy of application to textile-adhered bacteria. While such tests could be highly dependent on the experimental procedures, rarely has it been discussed for different analytical procedures applied for fibrous substrates. Microorganisms adhered on flat smooth surfaces, such as PET films, can be rather easily quantified through existing methods, such as labeling, staining, or detaching the bacteria for CFU counting or optical density measurements, whereas porous, irregular substrates, such as PP meltblown and PET spunbond, require more intricate techniques for accurate quantification. In this paper, three different evaluating methods of bacteria adhered on polymeric substrates were examined for their relevancy and limitations.

In the extraction method, the adhered bacteria were measured by detaching the cells from the substrates with the help of surfactant, enzyme and mechanical forces. For substrates with little pores or flat smooth surfaces, such as PET film, the extraction method can give quite an accurate result; however, for fibers surfaces with pores and multi-layered fibers, such as PET spunbond and PP meltblown, the extraction method could be misleading as the complete extraction through inner layers is not guaranteed. The colorimetric analysis with INT staining of bacteria may provide a better analytical solution for porous, multi-layered surfaces, as the method did not require the detachment of cells from surfaces. The formazan produced by the reaction of INT and bacteria was effectively separated by the DMSO extraction, and the amount of bacteria can be visually observed by the absorbency of colorant. The microscopic analysis with the image processing enabled the visual observation on bacterial distribution over the area, yet accurate quantification was not possible for fibrous surfaces. To conclude, the INT colorimetric method was believed to be a reliable and reproducible technique for quantification of adhered bacteria to fibrous substrates, as the bacterial presence is conveniently evidenced by the formation of formazan, avoiding the cell detachment from the porous textiles. Since the INT salt only reacts with live cells, the method can be used to evaluate the biocidal effects of antimicrobial textiles. 

Developing a proper quantification method for textile-adhered bacteria requires a collective understanding of both bacteria and material. The result of this study is expected to help advance our understanding of measurement technique for the bio-adhesive properties of variety of surfaces. Methods optimized for *E. coli* may not be optimal for other bacteria, but the overall methodological procedures and verification processes can be commonly applied. By analyzing different test methods, this study aims at broadly impacting the research in the field of anti-biofouling surfaces. Through the comparative evaluation of experimental procedures, this study is anticipated to help choosing a suitable evaluation method that can efficiently quantify the textile-adhered bacteria. Employing the relevant method, the next step of studies, with the extended scope of textile materials and bacteria type, are suggested for further analysis of interaction between the cell and the substrate chemistry.

## Figures and Tables

**Table 1:** Film and Nonwoven Substrates. PET = polyethylene terephthalate; PP = polypropylene.

Substrate	Material	Thickness (mm)	Basis Weight (g/m^2^)	Solidity (Unitless)	Porosity (%)	Mean Fiber Diameter (μm)
**PET-F**	PET film	0.05 (± 0.03, n = 3)	49 (± 3, n = 3)	-	-	NA
**PET-SB**	PET spunbond	0.15 (± 0.01, n = 3)	27 (± 1, n = 3)	0.02	98	19.8 (± 0.8, n = 15)
**PP-MB**	PP meltblown	0.25 (± 0.02, n = 3)	28 (± 1, n = 3)	0.02	98	1.4 (± 1.1, n = 15)

**Table 2:** Estimation of Bacteria-adhered Area Fraction on PET-F.

Surface	Pixel Count
PET-F surface (414 μm × 591 μm)	258,400
PET-F covered with bacteria	14,739
Percentage of surface area covered with bacteria	5.7%

**Table 3:** Comparison of Methods.

Method	Relevancy	Limitation
Extraction	Optical density of cell suspension linearly predicts the CFU. Count both live and dead cells.	Complete detachment of bacteria is not assured, especially for porous substrates.
Colorimetric	Cell detachment procedure is not necessary. Visualization of bacterial presence is possible, even in the naked eye. Count only the live cells.	Window for accurate quantification is narrow. Observation by the naked eye is difficult at the low level of bacterial concentration.
Microscopy	Direct observation on bacterial distribution on surfaces is possible. Only the topmost surface is observable.	Quantification is not accurate. Observation in depth layer is not possible.
